# Aerial Wildlife Image Repository for animal monitoring with drones in the age of artificial intelligence

**DOI:** 10.1093/database/baae070

**Published:** 2024-07-23

**Authors:** Sathishkumar Samiappan, B. Santhana Krishnan, Damion Dehart, Landon R Jones, Jared A Elmore, Kristine O Evans, Raymond B Iglay

**Affiliations:** Geosystems Research Institute, Mississippi State University, 2 Research Blvd, Starkville, MS 39759, United States; Geosystems Research Institute, Mississippi State University, 2 Research Blvd, Starkville, MS 39759, United States; Geosystems Research Institute, Mississippi State University, 2 Research Blvd, Starkville, MS 39759, United States; Computer Sciences and Computer Engineering, University of Southern Mississippi, 118 College Drive, Hattiesburg, MS 39406, United States; Department of Wildlife, Fisheries, and Aquaculture, Mississippi State University, Stone Blvd, Mississippi State, MS 39762, United States; Department of Wildlife, Fisheries, and Aquaculture, Mississippi State University, Stone Blvd, Mississippi State, MS 39762, United States; Geosystems Research Institute, Mississippi State University, 2 Research Blvd, Starkville, MS 39759, United States; Department of Wildlife, Fisheries, and Aquaculture, Mississippi State University, Stone Blvd, Mississippi State, MS 39762, United States; Department of Wildlife, Fisheries, and Aquaculture, Mississippi State University, Stone Blvd, Mississippi State, MS 39762, United States

## Abstract

Drones (unoccupied aircraft systems) have become effective tools for wildlife monitoring and conservation. Automated animal detection and classification using artificial intelligence (AI) can substantially reduce logistical and financial costs and improve drone surveys. However, the lack of annotated animal imagery for training AI is a critical bottleneck in achieving accurate performance of AI algorithms compared to other fields. To bridge this gap for drone imagery and help advance and standardize automated animal classification, we have created the Aerial Wildlife Image Repository (AWIR), which is a dynamic, interactive database with annotated images captured from drone platforms using visible and thermal cameras. The AWIR provides the first open-access repository for users to upload, annotate, and curate images of animals acquired from drones. The AWIR also provides annotated imagery and benchmark datasets that users can download to train AI algorithms to automatically detect and classify animals, and compare algorithm performance. The AWIR contains 6587 animal objects in 1325 visible and thermal drone images of predominantly large birds and mammals of 13 species in open areas of North America. As contributors increase the taxonomic and geographic diversity of available images, the AWIR will open future avenues for AI research to improve animal surveys using drones for conservation applications.

**Database URL**: https://projectportal.gri.msstate.edu/awir/

## Introduction

Conservation of animal species relies on the accurate quantification of their populations. The use and popularity of drones (unoccupied aircraft systems) to survey animals have increased exponentially in the last decade due to lower costs, increased count accuracy, improved efficiency, and lower risk of human injury compared to ground or other aerial surveys [1–4]. Despite these advantages, accurate identification of animals from drone imagery is challenging and time-consuming because surveys produce a large number of images to process for subsequent detection and counting of animals when conducted by humans [4, 5]. However, automated animal detection and identification using machine learning and artificial intelligence (AI) can substantially reduce time, bias, and logistical costs of processing images from drone surveys [3, 6–11].

The relative importance of algorithms versus training data to achieve highly accurate object recognition varies depending on the specific problem. There is a consensus among the research community that acknowledges the potential of superior algorithms to enhance image/object recognition capabilities over the quality and size of the training data. However, algorithm performance is intrinsically linked to the quality and representativeness of the training data [12]. So, even the most sophisticated algorithms can fail if the data which they learn from are not enough or do not accurately mirror real-world scenarios.

The evolution of benchmark datasets has been critical for driving the development of object recognition models. Early datasets like CIFAR (2009) [13] and PASCAL VOC (2005) [14, 15], while limited in scale, served as crucial catalysts for research, sparking interest among researchers (over 4600 mentions on Google Scholar as of 2024), and algorithmic advancements that would not have been possible otherwise. The release of COCO in 2014, with its larger and more diverse image collection of over 300 000 images and 80 classes, further enabled researchers to develop improved and complex image recognition models [16]. These foundational datasets inspired a large ImageNet dataset with over 14 million images that was released in 2010 [17]. The impact of ImageNet on object recognition accuracy is a great motivation to develop a new repository. Around 2010, at ImageNet’s release, the best models were only able to achieve a top-5 accuracy of 72.5%. By 2012, the top-5 accuracy had already improved to 83.6% with the introduction of the AlexNet deep learning model, and by 2022, state-of-the-art models have achieved top-1 accuracy exceeding 90%. This trajectory clearly demonstrates the transformative power of comprehensive training data for image recognition tasks.

A comprehensive review of existing computer vision datasets reveals a significant gap in resources tailored for aerial wildlife monitoring and management. Yet Another Computer Vision Index to Datasets (YACVID), an online database cataloging frequently used datasets in the field [18], currently lists over 530 entries (as of 2024). However, only two datasets within YACVID include wildlife imagery, one of which contains fewer than three avian species. Zooniverse provides several citizen science projects to identify animals [19]; however, very few are from an aerial perspective [20], and this site is not a permanent repository for storing images indefinitely. Thus, few of the documented datasets offer imagery of wildlife captured from an aerial perspective. Given the growing accessibility and affordability of small drones and their potential applications to wildlife research and conservation, the development of a specialized dataset focused on aerial wildlife imagery is imperative for fully utilizing the potential of these low-altitude sensing platforms.

Automated detection and classification of animals with AI have been successfully and widely implemented for images captured by camera traps, which also produce large number of images used to survey wildlife at close range (typically 10 to <1 m) for terrestrial and arboreal species [21, 22]. Researchers have populated open-access or other databases for the purpose of training machine learning approaches to accurately classify animals from camera trap images [22, 23], including Snapshot Serengeti for African mammals (>1.2 million images) and North American Camera Trap Images for birds and mammals (>3.7 million images). Such repositories successfully curate benchmark datasets ranging from hundreds to millions of images that researchers worldwide have used to train and test AI algorithms to standardize results and compare accuracy against previous studies for myriad applications (e.g. [17]) and to advance specific fields, including wildfire detection [24], diagnosing agricultural problems [25], and underwater image enhancement [26]. However, to date and to our knowledge, no standing repository exists for drone images, despite the need for automated classification of animals from drone images to increase the effectiveness of surveys for conservation applications [1, 6].

**Figure 1.**
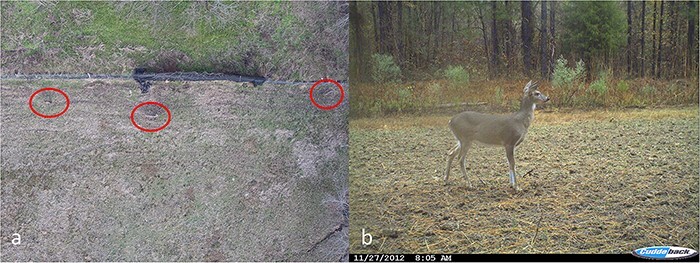
Images of white-tailed deer (*Odocoileus virginianus*) taken from an aerial perspective using a drone at 26 m above the ground level, with individuals circled in (a) and taken from a camera trap near the ground level (b).

Drone imagery poses additional challenges for accurate animal identification and classification compared to camera trap and other images, necessitating a dynamic, standing repository of images for animal classification training. First, animals are typically much farther from the sensor in drone images, depending on drone altitude (Fig. 1a); thus, animals appear smaller and consist of fewer pixels compared to their surroundings versus camera trap images (Fig. 1b; [6, 23]). Small animals provide less information (unique features such as texture and shape) for machine learning algorithms to successfully learn and classify accurately in test images [6, 27]. Second, the background in sequential drone images during aerial surveys often changes, whereas the background remains consistent across a trapping session for a camera trap [3, 6, 21]. Thus, machine learning algorithms can learn the static background for an individual camera trap and more easily detect differences between animals present or absent compared to ever-changing drone images [6, 27]. Finally, camera angles can vary in drone imagery, creating different views and shapes for animals compared to profile views of camera trap images [28]. Drone images are typically captured from nadir, near 90°, or at a variety of angles above animals [28]. Thus, animals in drone images may be less distinct from above compared to profile camera trap images (Fig. 1), providing potentially less or more variable information for training classification algorithms with drone images. For these reasons, AI classification algorithms require more drone images for successful training and implementation than camera trap studies, making the need for a drone image repository crucial for conservation applications.

The lack of annotated animal imagery for training is a critical bottleneck in achieving accurate performance with automated detection and classification of wild animals from drone surveys, including images at different altitudes, multiple camera angles and animal postures, complex backgrounds, occlusion of animals by various objects, and in diverse lighting conditions [1, 6, 27]. To fill this drone imagery gap and help advance and standardize automated animal classification, we created the AWIR(https://projectportal.gri.msstate.edu/awir/), a dynamic, interactive repository and database housing annotated images captured by drone users. We envision the AWIR as a collaborative resource for drone and computer vision researchers worldwide.

## Material and methods

The AWIR is a database environment serving two main purposes. First, the AWIR provides a repository to curate drone images of animals, similar to databases for other wildlife media [29]. Users can upload, annotate, and store animal imagery acquired from drones in an open-access environment available to the public. Second, the AWIR provides annotated imagery and benchmark datasets that are available for download by users to train AI algorithms to automatically detect and classify animals and compare algorithm performance, similar to other repositories [17].

### Downloading datasets

Image datasets are open access and available for direct download as ZIP files at https://scholarsjunction.msstate.edu/gri-publications/2/. We use the COCO format (JSON) outputs for each bounding box with a label.

### Uploading datasets

Users are encouraged to submit images with Exchangeable Image File Format (EXIF) content along with the source image file. EXIF content supplies informative metadata including camera settings, drone platform parameters, date, time, and location. Quality control for uploaded images is conducted by an AWIR administrator at Mississippi State University.

### Image annotation and quality control

**Figure 2.**
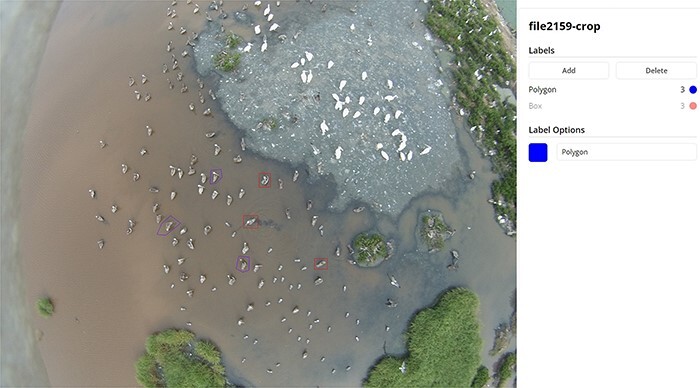
Labels of an image of brown pelicans (*Pelecanus occidentalis*) mixed with other species in the AWIR, annotated with bounding boxes as rectangular boxes or points defining polygons.

The AWIR annotation tool permits users to create bounding boxes and labels for animal objects in respective images. These functionalities are implemented to be intuitive and user-friendly. Bounding boxes can be drawn around each animal object directly using the provided software as either a box (user-defined rectangle) or polygon, bound by custom points placed by the user (Fig. 2). For both geometries, boxes or polygons are drawn to outline the animal object as closely as possible to eliminate or reduce background areas not associated with the animal object, which may add unnecessary variation for animal classification in computer vision algorithms. For polygons, the number of points required is as many as needed to adequately outline the object while excluding background pixels. A tool to redact sensitive, personally identifiable, or non-essential information is also provided.

The AWIR system employs a rigorous, standardized image annotation process to ensure data quality and consistency. Users are provided with detailed guidelines specifying the inclusion/exclusion criteria for animal objects during annotation, the appropriate level of precision for bounding boxes, and procedures for handling occlusion or ambiguous cases. To minimize annotator bias, each image is verified by administrators, and discrepancies are resolved and finalized. Prior to inclusion in the repository, all uploaded images undergo a multistep quality control process. This includes an initial check for file integrity and format compliance, followed by a review by administrators (authors of this manuscript). The submitted images are assessed for resolution, focus, exposure, and relevance to the AWIR’s scope. Additionally, the metadata associated with each image is verified for accuracy and completeness. Any images that fail to meet the established quality standards are returned to the submitter with feedback and recommendations for improvement.

### Functionality

The AWIR features additional challenges presented on the leaderboard page and uploaded by administrators to improve the AI algorithms for automatic detection and classification of animals in drone imagery, similar to other database efforts [17]. Users can download available datasets for each challenge and use them as benchmarks to test the performance of their respective algorithms. The leaderboard displays the username, accuracy percentage (animals correctly classified), and rank based on the performance in each challenge. One challenge provided is a dataset used to validate the performance of an algorithm in detecting and classifying three mammal species based on the fusion of both visible and thermal images [9]. Additional challenges based on benchmark datasets will be added as users upload images to the repository. The goal of the ongoing image classification challenge and the leaderboard is to motivate researchers to continually develop and improve models for the classification of drone images.

## Results

**Figure 3.**
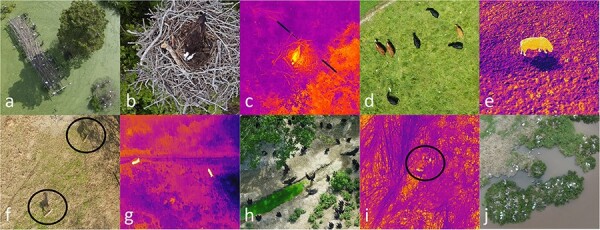
Images illustrating the diversity of mammalian and avian species in visible (RGB) and thermal images captured from drones available in the AWIR, including great egrets (*Ardea alba*, a), nesting osprey (*Pandion haliaetus*) visible (b), and thermal images (c), cattle egrets (*Bubulcus ibis*) and domestic cattle (*Bos taurus*) visible (d) and domestic cattle thermal images (e), white-tailed deer (*Odocoileus virginianus*) visible (f) and thermal images (g), black vultures (*Coragyps atratus*, h), thermal images of Northern bobwhite (*Colinus virginianus*) coveys (i), and nesting colonies of brown pelicans (*Pelecanus occidentali*s, j). Selected individuals are circled.

The AWIR presently contains 6587 animal objects in 1325 images among 13 species, mainly large animals in open areas, including 3 mammal species (78.0% of images and 63.2% of objects) and 10 bird species (22.0% of images and 36.8% of objects). Wild animals in images are from North America (100% of images and objects), but the dataset also includes captive white-tailed deer (*Odocoileus virginianus*, 192 images and 292 objects) and domestic species, including cattle (*Bos taurus*, 607 images and 3171 objects) and horses (*Equus caballus*, 234 images and 700 objects, Table 1, Fig. 3). Visible images (Red, Green and Blue (RGB)) represent 67.2% of the dataset (80.3% of objects), and thermal images comprise the remaining images (32.8% and 19.7% of images and objects, respectively, Table 1, Fig. 3). As contributions to the AWIR increase, we envision an increasing diversity of taxa, geographies, and image metadata.

**Table 1.** The number of images and objects (individual animals) in visible and thermal images of animal species taken from drones in the AWIR

		Visible		Thermal	
Species	Scientific name	Images	Objects	Images	Objects
Domestic cattle	*Bos taurus*	439	2478	168	693
Domestic horse	*Equus caballus*	162	497	72	203
White-tailed deer (captive)	*Odocoileus virginianus*	96	146	96	146
Cattle egret	*Bubulcus ibis*	60	170	58	102
Canada geese	*Branta canadensis*	62	82	0	0
Red-tailed hawk	*Buteo jamaicensis*	12	12	0	0
Osprey	*Pandion haliaetus*	16	23	14	16
Northern bobwhite	*Colinus virginianus*	0	0	27	140
Brown pelican	*Pelecanus occidentalis*	8	366	0	0
American white pelican	*Pelecanus erythrorhynchus*	6	77	0	0
Laughing gull	*Leucophaeus atricilla*	8	81	0	0
Black vulture	*Coragyps atratus*	11	864	0	0
Great egret	*Ardea alba*	10	491	0	0

## Discussion

Improving algorithms for the automated identification and classification of animals compared to other objects and from an aerial compared to a ground perspective requires (i) a larger image dataset and (ii) a larger diversity of images for each species in different geographic locations across multiple backgrounds, altitudes, angles, and poses. The AWIR provides the first open-source, collaborative database as a solution to overcome these challenges in two important ways to ultimately improve the efficiency of drone surveys. First, the AWIR provides a database platform for drone users to share their respective images and contribute to a larger pool of images to enhance the larger diversity of taxa and regions across different background and flight variables. Second, the AWIR provides annotated benchmark datasets that computer vision scientists can create or download to test the performance of their algorithms in a standardized way, resulting in improvements in automated identification and classification of animals from drone images. Additionally, the leaderboard acts as a publicly available scoreboard to encourage other researchers to improve their algorithms using respective benchmark datasets. Several studies within the last decade have demonstrated the utility of machine learning through AI to improve drone surveys through automatic detection and classification of predominantly large mammals and birds in open environments from drone images [5, 10, 11, 30, 31]. However, we anticipate that as the use of the AWIR increases, this trend will increase substantially on a more regular basis, similar to image repositories for animals captured by camera traps and other objects [16, 17, 22, 23], resulting in continual advances in performance for automated identification and classification of animals from drone images.

Although the number of images, taxonomic, and geographic coverage for the AWIR is limited at the time of this publication, two studies demonstrate the utility of images provided in our database. Zhou *et al*. [8] optimized combinations of epochs and percent training samples among three algorithms to achieve >99% overall accuracy in classifying cattle (*B. taurus*), horses (*E. caballus*), Canada geese (*Branta canadensis*), and white‐tailed deer (*O. virginianus*) taken from drones using only 100 images per species. Krishnan *et al*. [9] demonstrated that fusion of limited visible and thermal image pairs (<70 images per species) from drones provided improved detection and classification accuracy of cryptic white-tailed deer compared to visible or thermal images alone, as well as compared to more conspicuous domestic cattle and horses. These studies demonstrate how AI can be used for automating animal detection and classification from limited drone imagery of animals available in the AWIR, similar to larger databases for other types of images [17]. Furthermore, these datasets are available as benchmark datasets in the AWIR for future testing and performance improvement of computer vision algorithms. Thus, at its inception, the AWIR has already demonstrated its utility in improving AI applications.

### Future opportunities and applications

The AWIR is the first open-access database to curate, upload, and download animal images collected by drone platforms. The AWIR provides an opportunity for researchers using drones to study animals to pool resources and increase image availability on a global scale. A review of the literature on surveying animals with drones up to early 2022 revealed 216 studies of 285 species, primarily birds and mammals, in 46 countries [1]. Thus, the available global and taxonomic pool of animal imagery from drones is considerable and will likely grow exponentially as published studies on this topic continue to accelerate [1]. Pooling these resources across research groups in the AWIR provides unique opportunities to facilitate worldwide collaboration and new research avenues otherwise unknown to single groups. The annotation tools in the AWIR can also reduce the barriers to sharing images by redacting private or sensitive information. Additionally, for drone studies where animal images represent a portion of the data, the AWIR can provide a repository to store these data, as many peer-reviewed scientific journals require no-cost public accessibility of collected datasets as a condition of publication.

The AWIR also provides opportunities for researchers in computer vision to test the performance of new AI algorithms in benchmark datasets for better detecting and classifying animals of multiple taxa and geographies using drone images. These algorithms can also be shared in the AWIR making them available for wildlife applications among end users who are not computer scientists. Acquiring a variety of images for a given species that includes multiple flight altitudes, camera perspectives, backgrounds, behavioral poses, with and without shadows, occluded or fully visible, etc., can improve both detection and classification [17]. Additionally, improving the number and diversity of thermal images can improve detection and classification of nocturnal or cryptic species against their respective backgrounds [9–11]. As diverse images become available, future avenues for AI research to improve drone surveys include training algorithms to find ectothermic or small animals, occluded animals within landcover types with overhead vegetation coverage, and specific behaviors among a range of behaviors. The AWIR can facilitate future research as a collaborative environment to store, annotate, and test datasets of animal images to improve drone surveys for wildlife conservation.

## Data Availability

The AWIR and all its data will remain publicly available for a minimum of 2 years beginning from the day of publication.
